# Representation of Investigators by Gender Among Authors of Phase 3 Oncology Trials Worldwide

**DOI:** 10.1001/jamanetworkopen.2022.0031

**Published:** 2022-02-25

**Authors:** Maishara Muquith, Tri Pham, Magdalena Espinoza, David Hsiehchen

**Affiliations:** 1 University of Texas Southwestern Medical School, Dallas; 2 Division of Digestive and Liver Diseases, Department of Internal Medicine, University of Texas Southwestern Medical Center, Dallas; 3 Division of Hematology and Oncology, Department of Internal Medicine, University of Texas Southwestern Medical Center, Dallas

## Abstract

This cross-sectional study uses worldwide oncology trial data to examine the proportion of female investigators in each country and across all trials.

## Introduction

Despite an increasing number of women in medicine, gender disparities in academic medicine are apparent in authorship, leadership positions, and funding.^1,2,3,4^ Clinical trial research is important for scientific advancement and the professional development of oncology investigators. We examined the prevalence of female investigators and their recognition as authors of phase 3 randomized clinical trials (RCTs) because phase 3 trial publications do not typically include all investigators as authors.

## Methods

In this cross-sectional study, we analyzed all phase 3 oncology RCTs published in 3 journals (*New England Journal of Medicine*, *Lancet*, and *Lancet Oncology*) between January 2019 and December 2020 that listed full investigator names and country affiliations. Per the Common Rule (45 CFR §46), institutional review board approval was not sought because this study is not human participant research. This study follows the Strengthening the Reporting of Observational Studies in Epidemiology (STROBE) reporting guideline for cross-sectional studies.

We excluded publications reporting secondary analyses. Gender was determined using genderize.io if the probability of the assigned gender was greater than 0.7. Otherwise, Google searches of the name and affiliation were conducted, and gender was assigned according to professional or social profiles. Authors included persons included in the byline of articles. Investigators and steering committee members were identified from investigator and committee rosters, respectively. The primary outcome measure was the proportion of female investigators in each country and proportion of female authors across the trials. The proportion of female physicians was abstracted from the most recent available data (2015-2019) for each country from the World Health Organization Global Health Workforce Statistics database. Differences in proportions were tested using 2-sided χ^2^ tests, with significance set at *P* < .05. Data were analyzed using SPSS version 24 (IBM). Data were analyzed from March 2021 to May 2021.

## Results

Across 114 oncology RCTs, gender was identified for 15 805 of 15 915 investigators, 2354 of 2753 authors who were investigators, and all 64 steering committee members. The proportion of female investigators was 27.6% (4363 women), while the proportion of female investigator authors (of all investigator authors) was 22.9% (540 women) (odds ratio, 0.78; 95% CI, 0.70-0.86) (Table). There was a lower proportion of female steering committee members (13 women [20.3%]), female first authors (25 women [21.9%]), and female last authors (22 women [19.3%]) compared with the proportion of female investigators. Across cancer types, women represented a minority of investigators and even smaller proportions of all authors, first authors, and last authors (Table). There were 399 authors who were not investigators, including industry authors, statisticians, and other research personnel, 230 of whom were women (57.6%).

**Table.** Proportion of Investigators, Steering Committee Members, or Authors Who Are Women in Oncology Trials Across Cancer Types

Cancer type	Women, No. (%)				
	Investigators^a^ (N = 15 805)	Committee members^b^ (n = 64)	Authors^c^ (n = 2354)	First authors (n = 114)	Last authors (n = 114)
All	4363 (27.6)	13 (20.3)	540 (22.9)	25 (21.9)	22 (19.3)
Aerorespiratory	679 (23.3)	2 (20.0)	75 (19.5)	6 (30.0)	3 (15.0)
Blood	706 (29.5)	4 (44.4)	95 (22.7)	4 (22.2)	2 (11.1)
Breast	1220 (41.0)	4 (33.3)	78 (33.1)	5 (35.7)	6 (42.9)
Gastrointestinal	404 (27.7)	2 (16.7)	76 (22.3)	3 (21.4)	2 (14.3)
Genitourinary	718 (16.8)	1 (4.8)	93 (16.6)	2 (6.9)	7 (24.1)
Gynecological^d^	413 (37.9)	NA	65 (34.2)	3 (37.5)	0
Skin^d^	109 (27.3)	NA	13 (18.8)	2 (28.6)	1 (14.3)
Other^d^	114 (38.0)	NA	45 (29.2)	0	1 (25.0)

Abbreviation: NA, not applicable.

^a^ Investigators included all persons listed in the investigator roster in the data supplement of publications.

^b^ Committee members included all persons listed in the committee roster in the data supplement of publications.

^c^ Authors included all investigators included in the byline of publications.

^d^ Committee member rosters were not available for gynecological, skin, and other cancer type trials.

Among 52 countries with at least 10 investigators identified in this study, nearly all demonstrated a greater proportion of physicians who were women than the proportion of investigators who were women (Figure). There was a significant correlation between female investigators and physicians (*r* = 0.34; *P* = .01). However, the correlation coefficient of 0.34 indicates that only 11.5% of the differences in the proportion of investigators that are women between countries is explained by the proportion of physicians that are women.

**Figure.**
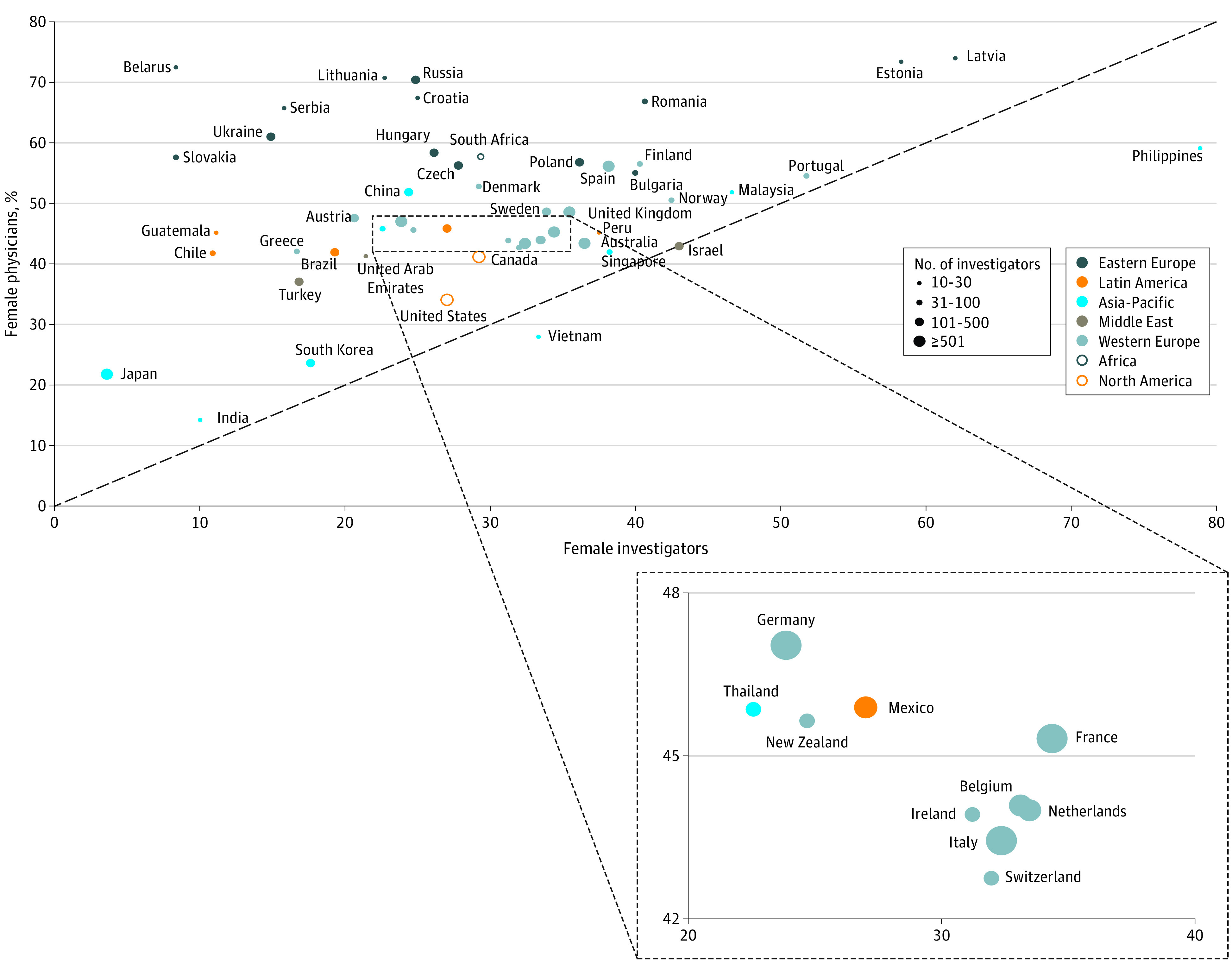
Association Between Female Investigators and Physicians Across Countries Scatterplot depicts the association between the proportions of female investigators and physicians across 52 countries. The size of bubbles represents the number of investigators, and the color of bubbles represents geographical regions. The dashed diagonal line indicates an equal proportion of female investigators and physicians. Countries lying above the line have a lower proportion of female investigators than physicians compared with countries below the line.

## Discussion

Prior studies^5,6^ found a low prevalence of female authors on trial publications without addressing the representation of women among investigators and neglected country-specific proportions of female physicians. The findings of this cross-sectional study show that women are globally underrepresented as investigators in phase 3 oncology RCTs and are even less likely to be recognized as authors, designated as first or last authors, or present on steering committees. However, the degree of underrepresentation varied widely across nations. Further research is needed to identify specific and targetable barriers to the participation and recognition of women as investigators in oncology trials.

A limitation of this study is that gender was identified for most but not all investigators. Given the lack of data on the proportion of female oncologists by country, we used workforce data provided by the World Health Organization, which is a definitive source for cross-country data comparisons. Other limitations include the limited representation of countries in the trials assessed and the exclusion of early-phase trials.
